# Comparative Embryonic Spatio-Temporal Expression Profile Map of the *Xenopus* P2X Receptor Family

**DOI:** 10.3389/fncel.2019.00340

**Published:** 2019-07-26

**Authors:** Camille Blanchard, Eric Boué-Grabot, Karine Massé

**Affiliations:** ^1^Université de Bordeaux, Institut des Maladies Neurodégénératives, UMR 5293, Bordeaux, France; ^2^CNRS, Institut des Maladies Neurodégénératives, UMR 5293, Bordeaux, France

**Keywords:** P2 receptors, purinergic signaling, embryogenesis, neurogenic placodes, sensory neurons, nervous system, *Xenopus laevis*

## Abstract

P2X receptors are ATP-gated cations channels formed by the homo or hetero-trimeric association from the seven cloned subunits (P2X1-7). P2X receptors are widely distributed in different organs and cell types throughout the body including the nervous system and are involved in a large variety of physiological but also pathological processes in adult mammals. However, their expression and function during embryogenesis remain poorly understood. Here, we report the cloning and the comparative expression map establishment of the entire P2X subunit family in the clawed frog *Xenopus*. Orthologous sequences for 6 mammalian P2X subunits were identified in both *X. laevis* and *X. tropicalis*, but not for P2X3 subunit, suggesting a potential loss of this subunit in the *Pipidae* family. Three of these genes (*p2rx1, p2rx2*, and *p2rx5*) exist as homeologs in the pseudoallotetraploid *X. laevis*, making a total of 9 subunits in this species. Phylogenetic analyses demonstrate the high level of conservation of these receptors between amphibian and other vertebrate species. RT-PCR revealed that all subunits are expressed during the development although zygotic *p2rx6* and *p2rx7* transcripts are mainly detected at late organogenesis stages. Whole mount *in situ* hybridization shows that each subunit displays a specific spatio-temporal expression profile and that these subunits can therefore be grouped into two groups, based on their expression or not in the developing nervous system. Overlapping expression in the central and peripheral nervous system and in the sensory organs suggests potential heteromerization and/or redundant functions of P2X subunits in *Xenopus* embryos. The developmental expression of the p2rx subunit family during early phases of embryogenesis indicates that these subunits may have distinct roles during vertebrate development, especially embryonic neurogenesis.

## Introduction

The nucleotide ATP, as the universal source of energy, is an essential intracellular molecule to the survival of cells and whole organisms. However, the discovery of the first P2 purinergic receptor at the beginning of the 1990s confirmed Geoffrey Burnstock’s hypothesis that ATP acts also as an extracellular signaling molecule (Burnstock, 2014). In the last 25 years, numerous studies demonstrated the fundamental importance of extracellular ATP in the physiology of all organs through the activation of ionotropic P2X and metabotropic P2Y receptors (Ralevic and Burnstock, 1998). Purinergic signaling pathway is not restricted to extracellular ATP actions. The metabolic breakdown of extracellular ATP by ectonucleotidases is a source of ADP and adenosine. Both ATP derivatives act as extracellular signaling molecule through the activation of P2Y and adenosine G-protein coupled receptors (Adora) respectively (Yegutkin, 2014). It is nowadays recognized that the purinergic signaling in various organs play important physiological roles but also in diseases, placing purinergic receptors and ectonucleotidases as theurapeutic targets (Burnstock, 2018). In particular, in the nervous system, ATP and adenosine are involved in neuromodulation, glial-neuron interaction, and sensory transmission but also in neuropathic pain, neurodegenerative diseases, and multiple sclerosis (Burnstock et al., 2011; Khakh and North, 2012; Burnstock, 2016, 2017; Boué-Grabot and Pankratov, 2017; Domercq et al., 2018). Although the purinergic signaling has received less attention in a developmental context, several recent studies have shown a role of extracellular ATP and its derivatives in progenitor cell proliferation, migration, differentiation and synaptogenesis (Del Puerto et al., 2013; Oliveira et al., 2016; Fumagalli et al., 2017; Rodrigues et al., 2018). Furthermore, the description of the ectonucleotidases and purinoreceptors embryonic expression profile *in vivo* provides indication of their implication during nervous system development (Massé and Dale, 2012).

Due to the high number of actors, their large and overlapping expression profile and the very broad range of the purinergic receptor sensitivities, deciphering *in vivo* the specific embryonic functions of each extracellular purine is quite challenging. We decided to tackle this by studying each member of purinergic receptors and ectonucleotidases family using the clawed frog *Xenopus* embryo due to its numerous advantages, e.g., large size, large number production, relative transparency, external development and rapid generation times. Moreover, as its developmental cycle is temperature dependent, it is possible to modulate the length of the early stages such a neurulation allowing to access easily to the embryos at all developmental stages. Although having a pseudo-allotetraploid genome, the recent development of *Xenopus* genetics has broadened the use of this model especially as animal model for human pathologies (Sater and Moody, 2017; Tandon et al., 2017). We have previously established the embryonic expression pattern of enpp and entpd ectonucleotidases and adenosine signaling pathway members in *Xenopus* embryos. The specific spatio-temporal expression profile of each member of ectonucleotidases and adenosine receptors, suggested that ATP, ADP and adenosine may have distinct and specific functions during vertebrate embryogenesis (Massé et al., 2006, 2010; Tocco et al., 2015). Indeed, we demonstrated that ADP, via the P2Y1 receptor, triggers eye development by regulating the expression of the eye field transcription factors, such as pax6 (Massé et al., 2007). *Xenopus* is therefore an ideal model to address the functional roles of the purinergic pathway during the early phases of vertebrate embryogenesis, phases that are difficult to study in other vertebrate models (Massé and Dale, 2012).

Here we characterized the *Xenopus* P2X receptor subunit family. P2X receptors are ATP-gated cation channels formed by homomeric or heteromeric association of three subunits, which are involved in numerous physiological and pathological processes (North, 2002; Khakh and North, 2006; North, 2016). Seven genes for P2X subunits have been cloned and shared a common topology with two membrane-spanning domains, a large extracellular domain containing the ATP binding sites and intracellular N and C termini (Kawate et al., 2009; Habermacher et al., 2016). We report the cloning of the entire family in *Xenopus* laevis and *tropicalis* and reveal the absence of the *p2rx3* gene in both of these species. Our phylogenetic data demonstrate a high degree of conservation of these *p2rx* sequences during vertebrate evolution. Analysis of the temporal and spatial expression of each amphibian subunit during *X. laevis* development showed that all identified subunits are expressed during development. Based on their expression or not in the nervous system, two groups of subunits can be proposed. Transcripts of all *p2rx* subunits, except *p2rx2.S* and *p2rx5.L*, are detected in the central and peripheral nervous system and their overlapping expression suggests potential hetero-oligomerization or redundant functions in the sensory system. A group of three subunits, *p2rx2.S, p2rx4.L, and p2rx5*, display specific expression in mesodermal derivatives suggesting these subunits may regulate renal and muscle development.

## Materials and Methods

### Bioinformatics

Sequences were identified on the NCBI and Xenbase databases. BLAST (Basic Local Alignment Search Tool) searches were performed on the NBCI Nucleotide and on the Xenbase *Xenopus laevis* 9.1 genome databases (Altschul et al., 1990). This identification was based on *p2rx* subunits orthologs nucleotide alignments, using either predicted (from automated computational analysis) mRNA sequences from *Xenopus* genomic sequences or validated mRNA sequences from different species such as *Homo sapiens*, *Mus musculus*, *Rattus norvegicus*, *Danio rerio*, *Gallus gallus*, *Takifugu rubripes*, *Xenopus tropicalis*, and *Xenopus laevis*. Conceptual translation of cDNA and protein sequence analysis was performed on the ExPASy internet site using the program Translate Tool^1^ (Artimo et al., 2012). Accession numbers of all sequences used in this study are given in Supplementary Table S1. Alignments were performed on the EMBL-EBI internet site using the program Pairwise sequence global alignment EMBOSS Needle and Multiple Sequence alignment Clustal Omega program (Needleman and Wunsch, 1970; Thompson et al., 1994). A phylogenetic tree was created on the Phylogeny.fr platform using muscle for multiple alignments, PhyML for tree building and TreeDyn for tree rendering (Dereeper et al., 2008).

### Embryos Culture

*Xenopus laevis* males and females were purchased from the CNRS *Xenopus* breeding Center (Rennes, France). Embryos were obtained by *in vitro* fertilization of eggs collected in 1X MMR (Marc’s Modified Ringers) saline solution [100 mM NaCl, 2 mM KCl, 2 mM CaCl_2_, 1 mM MgSO_4_, 5 mM Hepes (pH 7.8), pH 7.4], from a hormonally [hCG (Agripharm), 750 units] stimulated *X. laevis* female by adding crushed testis isolated from a sacrificed male. Fertilized eggs were dejellied in 3% L-cysteine hydrochloride, pH 7.6 (Sigma-Aldrich), and washed several times with 0.1X MMR. Embryos were then cultured to the required stage in 0,1X MMR in presence of 50 mμ/mL of gentamycin sulfate (Sigma-Aldrich). The embryos were staged according to the Nieuwkoop and Faber table of *X. laevis* development (Nieuwkoop and Faber, 1994).

### RT-PCR

RNA extraction from whole embryos and cDNA synthesis were performed as previously described (Massé et al., 2010). For each subunit, specific primers were designed, when possible, on two different exons with at least one primer positioned at exon junctions (Supplementary Table S2), in order to discriminate genomic from cDNA amplification. Furthermore, those chosen primers were selected to differentiate homeologs expression, with a restrictive annealing possibility on the other homeologous mRNA sequence. After optimization of the PCR conditions using a gradient PCR machine (Bio-Rad), PCR products were verified by sequencing (Beckman Coulter Genomics Company). To assess semi-quantitative PCR experiments, the quantity of input cDNA was determined by equalization of the samples with the constant gene *odc* (ornithine decarboxylase) (Bassez et al., 1990). Linearity of the signal was controlled by carrying out PCR reactions on doubling dilutions of cDNA. Negative controls (-RNA, -RT, and -cDNA) were also performed.

### *In situ* Hybridization

Whole-mount *in situ* hybridizations were carried out as previously described (Massé et al., 2006, 2010; Blanchard and Massé, 2019). In order to detect the expression of each *p2rx* subunit, fragments of their cDNA, except for *p2rx4*, were subcloned into plasmid pBSKS, as described in Supplementary Table S3, in order to generate specific antisense and sense probes. Riboprobes were generated by *in vitro* transcription using a DIG RNA labeling kit following manufacturer’s recommendations (Roche) after linearization of the corresponding plasmid (see Supplementary Table S3 for details). Templates were designed in order to generate 250–600 bases riboprobes; when using larger riboprobes, a limited alkaline hydrolysis was performed. Riboprobe hybridization detection was carried out with an anti-DIG Alkaline Phosphatase antibody (Roche, Reference 11093274910) and the BM-Purple AP substrate (Roche Reference, 11442074001). After bleaching, embryos were photographed using the SMZ18 binocular (Nikon).

### Embedding and Sectioning

After whole-mount *in situ* hybridization, embryos were gradually dehydrated in 100% gelatin/sucrose-PBS solution before an overnight incubation in 100% gelatin/sucrose solution. Identified embryos were embedded in Tissue-Tek OCT, frozen on dry ice and conserved at −80°C. The frozen blocks were sectioned on a cryostat at 10 μm thickness. Transverse sections were also performed with a razor blade on fixed embryos. Sections were photographed using the SMZ18 binocular (Nikon).

## Results

### Cloning of the Different *p2rx* Subunit Genes

The *p2rx* subunit sequences were identified by bioinformatics analysis, this work having benefited from the recent *X. tropicalis* and *X. laevis* genome sequencing (Hellsten et al., 2010; Session et al., 2016). The accession numbers of the protein sequences of the amphibian subunits can be found in Supplementary Table S1A. Unexpectedly, no genomic sequences were identified for *p2rx3* subunit in both *X. laevis* and *X. tropicalis* genomes.

All *Xenopus tropicalis* sequences are available on the Xenbase website, although only as annotated and predicted by automated computational analysis from genomic sequences. Their identity as P2X subunits was confirmed by conducting two complementary analyses: (1) Blastx search of these predicted *X. tropicalis* sequences on the human protein database (NCBI) was first conducted. (2) *X. tropicalis* cDNA sequences were deduced from the genomic sequences and corrected by reference to the human sequences according to the Breathnach and Chambon rule (Breathnach and Chambon, 1981). The protein sequences were deduced from conceptual translation and aligned with their potential orthologs.

Regarding the *Xenopus laevis p2rx* subunits, the *p2rx4.L* and *p2rx7.L* sequences have been previously published (Juranka et al., 2001; Paukert et al., 2002). *P2rx1.L, p2rx2.L, p2rx5.L, and p2rx5.S* sequences were available on the Xenbase site, but only as annotated sequences. Sequences verification was performed as described above for the *X. tropicalis* sequences. *P2rx1 and p2rx6 X. tropicalis* protein sequences blast on the *Xenopus laevis* protein database (NCBI website) allowed us to identify the *p2rx1.L* sequence and two *X. laevis* hypothetical proteins, whose sequences were derived from genome annotation. The identity of these sequences as *p2rx1.S* and *p2rx6.L* subunits was confirmed as described above. A *p2rx2.S* genomic sequence was identified but no cDNA or protein sequences were available on the Xenbase or NCBI website. From this genomic sequence, we retrieved the cDNA sequence after checking the exon/intron boundary by comparison to the human *p2rx2* ones according to the Breathnach and Chambon rule (Breathnach and Chambon, 1981) and carried out several blasts on *Xenopus laevis* databases (NCBI website). Alignment of these different sequences allowed us to generate the consensus and complete sequence of *p2rx2.S* subunit cDNA. The protein sequence was then obtained by conceptual translation and checked by blast on the protein databases (NCBI website).

### Evolutionary Conservation of P2X Subunit Receptors

In order to characterize the evolution of P2X receptor subunits in vertebrates, we performed an alignment of all *Xenopus* protein sequences (Supplementary Figure S1). In *X. laevis*, *p2rx1*, *p2rx2*, *p2rx5* have two homeologs (L and S), whose protein sequences display high degree of identity (more than 90%, Table 1A). However, the percentage of identity between the different *X.laevis* p2rx subunit sequences is less than 50% with p2rx4.L sharing the highest identity percentage with p2rx5 homeologs. The identity percentage between p2rx7.L and its *X. laevis* paralogs sequences is less than 30%. This low percentage is certainly due to the presence of the long and most divergent C terminus domain of p2rx7.L in this analysis.

**TABLE 1A:** Relatedness of the *X. laevis* p2x receptor subunits.

***X. laevis* protein**	**p2rx1.L**	**p2rx1.S**	**p2rx2.L**	**p2rx2.S**	**p2rx4.L**	**p2rx5.L**	**p2rx5.S**	**p2rx6.L**
p2rx1.S	91.7 (95.9)							
p2rx2.L	31.6 (50.9)	32.3 (53.6)						
p2rx2.S	31.8 (52.2)	32.8 (54.0)	94.1 (95.2)					
p2rx4.L	45.5 (64.2)	46.6 (63.7)	37.1 (55.9)	38 (56.9)				
p2rx5.L	43.6 (61.6)	42.8 (60.2)	37.4 (55.6)	39.1 (59.2)	46.9 (63.3)			
p2rx5.S	44.0 (61.7)	42.7 (59.9)	37.7 (56.0)	38.3 (58.1)	47.5 (63.2)	92.5 (96.1)		
p2rx6.L	34.5 (52.8)	35.4 (53.7)	34.0 (51.8)	33.7 (51.9)	42.4 (58.3)	44.5 (61.2)	45.9 (63.5)	
p2rx7.L	27.9 (40.0)	27.5 (39.5)	27.5 (39.5)	27.6 (40.9)	31.3 (45.0)	27.3 (40.8)	27.4 (40.5)	23.1 (35.9)

*The percentages of amino acid identity and similarity (in bracket) between the different p2rx proteins were determined by pairwise alignment using the Needleman–Wunsch global alignment on the EMBL-EBI website.*

**FIGURE 1:**
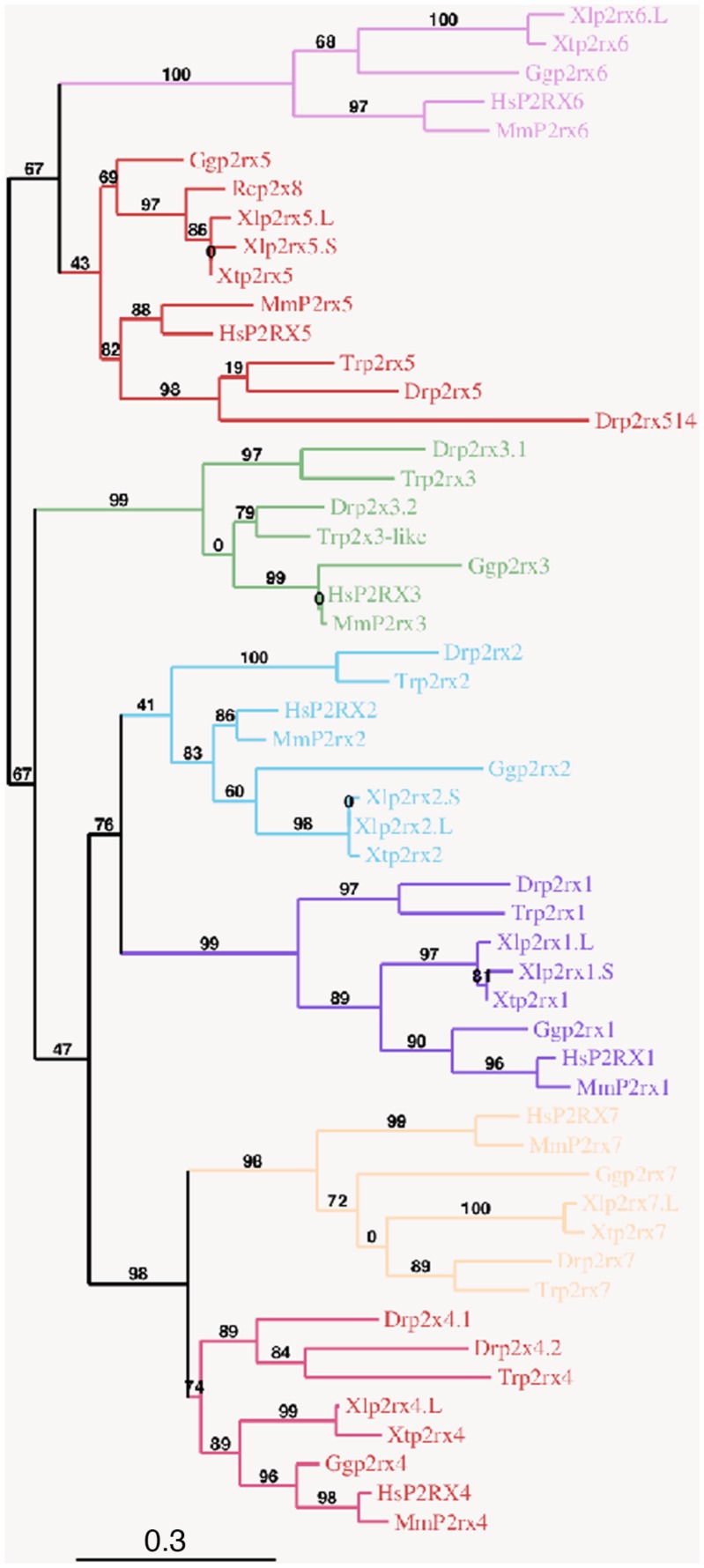
Phylogenetic conservation of the vertebrate p2x receptor subunits. A phylogenetic tree of the p2rx subunits family based on African clawed frogs (*Xenopus laevis*, Xl and *Xenopus tropicalis*, Xt), human (*Homo sapiens*, Hs), mouse (*Mus musculus*, Mm), chicken (*Gallus gallus*, Gg), zebrafish (*Danio rerio*, Dr), Japanese puffer (*Takifugu rubripes*, Tr) and American bullfrog (*Rana catesbeiana*, Rc) was constructed on the plastform phylogeny.fr, using the Maximum Likelihood method. Bootstrap percentages (ranging from 0 to 100) are indicated at each node of the tree while branch lengths are representative of sequence substitution rates. P2rx1, 2, 3, 4, 5, 6, 7 subunit subfamilies are respectively colored in purple, blue, green, dark pink, red, light pink, and orange. The Genbank accession numbers of the different receptors are given in the Supplementary Tables S1A,B except for Rcp2rx8 (Accession Number: AAL24075.1).

To address the evolutionary diversification of the P2X subunits in vertebrates, a phylogenetic analysis of P2X proteins was carried out (Figure 1). This resulting tree reveals a clear separation between the p2rx6 and p2rx5 proteins and the other p2rx family members, which could be subdivided into three groups, p2rx3 group, p2rx1 and p2rx2 group and p2rx4 and p2rx7 group. Each member is more related to its orthologs than to the other family members in the same species, suggesting that any function identified in *X. laevis* may well be conserved in other vertebrates (Table 1B). The percentage of identity between *Xenopus* and its orthologous proteins is between 44 and 76%, with p2rx7.L being the least conserved protein as it only shares 38–46% of identity with zebrafish and chick p2rx7 subunit respectively. P2rx4.L is the most conserved member during vertebrate evolution, with a percentage of identity higher to 49% with the other P2X4 vertebrate proteins.

**TABLE 1B:** Relatedness between the *X. laevis* p2x receptor subunits and their vertebrate orthologs.

		**orthologs**				
		***X. tropicalis***	***H. sapiens***	***M. musculus***	***G. gallus***	***D. rerio***
*X. laevis proteins*	p2rx1.L	94.4 (96.4)	59.6 (75.3)	59.3 (73.8)	60.8 (74.5)	51.6 (66.4)
	p2rx1.S	93.0 (97.1)	58.4 (76.4)	58.4 (75.7)	60.9 (76.3)	50.8 (67.1)
	p2rx2.L	96.8 (97.7)	48.1 (58.6)	50.5 (61.2)	48.4 (63.3)	44.3 (59)
	p2rx2.S	93.6 (95.0)	46.5 (56.9)	48.8 (58.9)	47.8 (62.7)	45.3 (60.6)
	p2rx4.L	91.0 (94.0)	66.2 (78.7)	66.6 (81.1)	68.9 (82.1)	56.2 (74.6)/ 49.8 (70.2)
	p2rx5.L	93.0 (97.1)	57.7 (72.3)	57.9 (71.9)	75.5 (85.5)	48.9 (61.4)
	p2rx5.S	93.2 (95.9)	57.5 (70.6)	57.5 (71.3)	76 (85.7)	50 (51.9)
	p2rx6.L	91.9 (95.6)	47.1 (60.5)	53.4 (67.3)	52.7 (67)	n.a
	p2rx7.L	81.6 (87.8)	45.7 (60.6)	45.1 (59.7)	46.6 (61.2)	38.4 (54.8)

*The percentages of amino acid identity and similarity (in bracket) between the p2rx proteins were determined by pairwise alignment using the Needleman–Wunsch global alignment on the EMBL-EBI website. The Genbank accession numbers are given in the Supplementary Table S1. No zebrafish p2rx6 subunit has been described (Kucenas et al., 2003). n.a, not applicable.*

### Temporal Expression of the p2rx Gene Family During *X. laevis* Development

**FIGURE 2:**
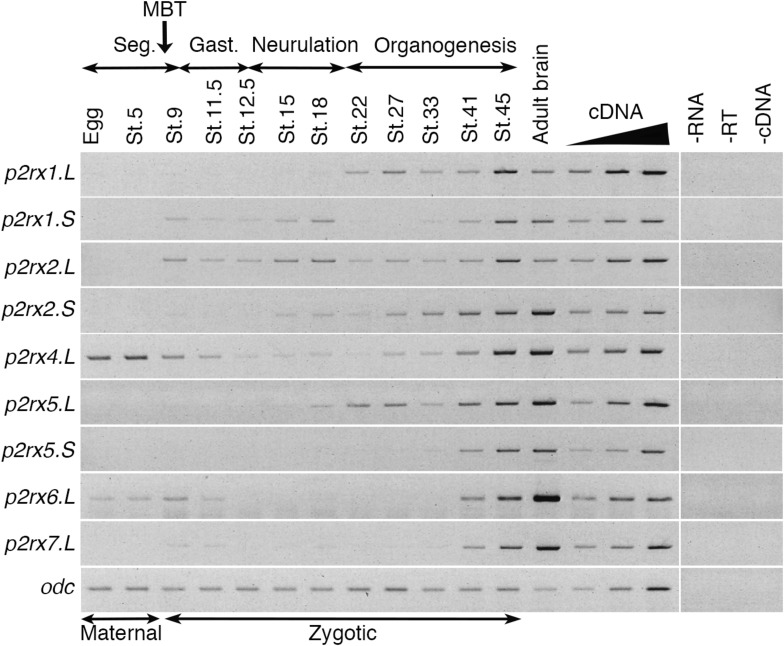
Temporal expression profile of *p2rx* genes during embryogenesis. RT-PCR analysis showing the expression pattern of *p2rx* subunits in *X. laevis* unfertilized egg and embryos at different stages (St.). Adult brain cDNA was used as positive control and three negative controls (-RT, -RNA, -cDNA) were also performed. The linearity of the signal was controlled by carrying out PCR reactions on doubling dilutions of cDNA from stage 45 embryo, illustrated by the triangle. *Odc* (*ornithine decarboxylase*) was used as a loading control. The different phases of embryogenesis and the Mid-Blastula transition (MBT), the switch between maternal and zygotic expression, are indicated above the different embryonic stages. Seg, segmentation; Gast, Gastrulation.

The temporal expression of the *p2rx* genes during embryonic development was assessed by RT-PCR (Figure 2). Adult brain cDNA was used as positive control. Each subunit displays a specific expression profile; however, the level of their expression increases during development to reach a maximum at stage 45, last embryonic stage tested during this experiment. The *p2rx1.L* subunit is expressed from late neurula stage whereas expression of its homeolog *p2rx1.S* is weakly detected from late segmentation to mid-neurulation and at a higher level during late organogenesis. The two *p2rx2* subunits display similar expression profile: both of these subunits are not expressed maternally and their zygotic expression can be detected from late segmentation stage, stage 9, although *p2rx2.L* seems more expressed during gastrulation. The *p2rx4.L* gene displays a specific expression profile, as being the only one subunit whose expression is detected at all stages tested, with a high level of maternal expression. This is in agreement with its previously published expression in oocytes (Juranka et al., 2001). The homeologs *p2rx5.L* and *p2rx5.S* are not maternally expressed. Zygotic expression of *p2rx5.L* is visible during neurulation whereas *p2rx5.S* expression is weakly detected at stage 33. Expression of these both homeologs is detected at a higher level from stage 41. The *p2rx6.L* subunit is the second *p2rx* gene being expressed maternally; however, its zygotic expression is only detected at late organogenesis stages. The *p2rx7.L* gene displays another distinct expression profile with its expression only clearly visible at late organogenesis stages, from stage 41, suggesting no functional roles for this subunit at early embryogenesis stages.

### Spatial Expression of the *p2rx* Gene Family During *X. laevis* Development

**FIGURE 3:**
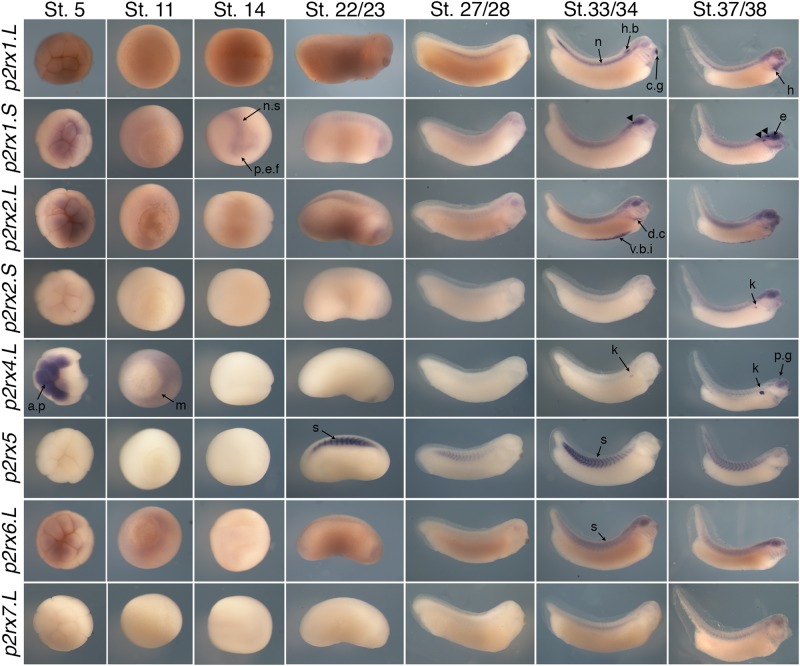
Comparative spatial expression profile of *p2rx* genes during embryogenesis. Whole-mount *in situ* hybridization with specific DIG-labeled antisense RNA probes was performed on embryos from stages (St.) 5 to 37/38. Representative embryos were photographed at the magnification X20 for stages 5, 11, and 14 and X16 for later stages. Stage 5: animal pole view, stage 11: vegetal pole view, stage 14: dorsal view except for *p2rx1.S* (anterior view), later stages: lateral views, with dorsal is up and anterior is right. The arrowheads indicate the staining in the neurogenic placodes and peripheral nervous system detailed in Figure 5. a.p, animal pole; c.g, cement gland; d.c, duct of Cuvier; e, eye; h, heart; h.b, hindbrain; k, kidney; m, mesoderm; n, notochord; n.s, nervous system; p.e.f, presumptive eye field; p.g, pineal gland; s, somite; t.n, trigeminal nerve; v.b.i, ventral blood island.

**FIGURE 4:**
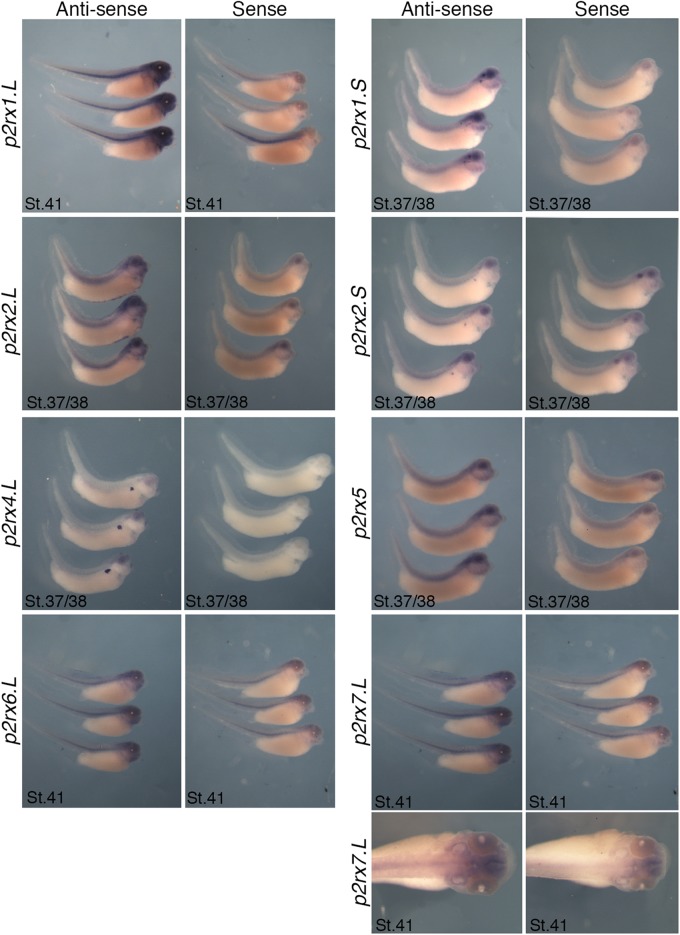
Comparative staining obtained with the antisense and sense probes of p2rx genes. Lateral views of three representative embryos at stage 37/38 or stage 41stained with the antisense and sense probes for *p2rx1.L, p2rx1.S, p2rx2.L, p2rx2.S, p2rx4.L, p2rx6.L*, and *p2rx7.L*. subunits and *p2rx5* homeologs. Some unspecific staining, due to probe trapping, can be observed in the head region (brain ventricles, otic vesicle) and in the notochord. For *p2r7.L* subunit, a dorsal view of a representative embryo completes the lateral views. For lateral views, dorsal is up and anterior is right. For dorsal views, anterior is up.

**FIGURE 5:**
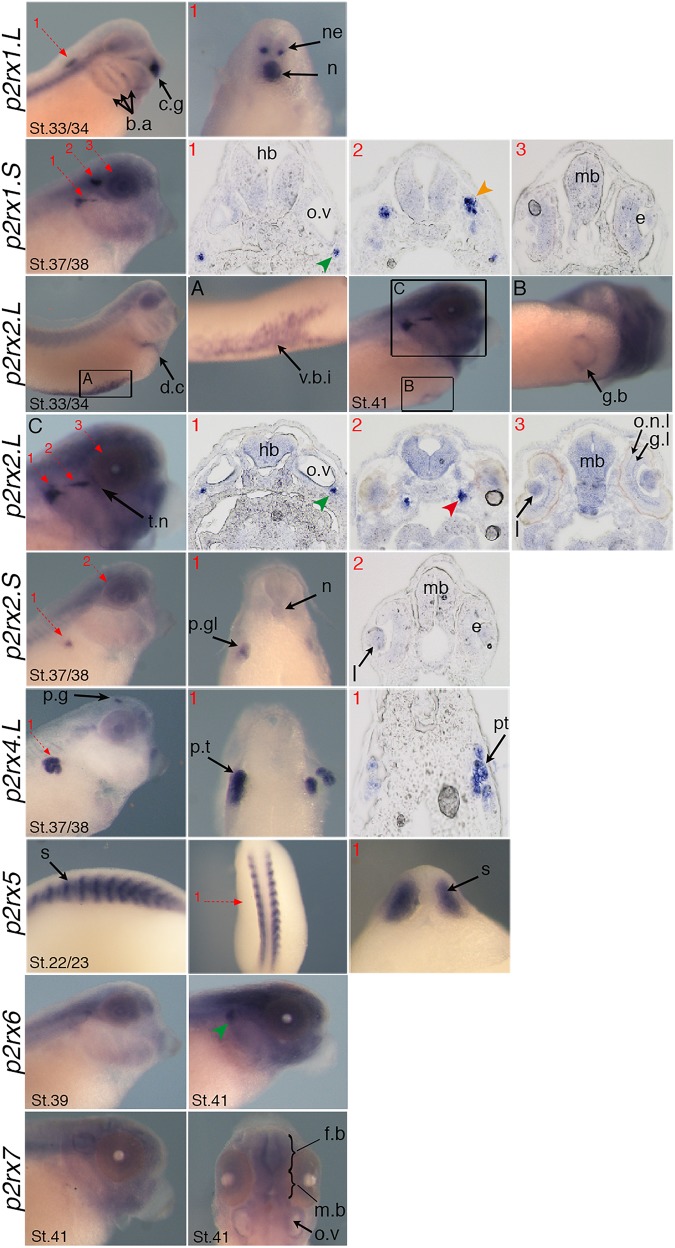
Specific expression domains of *p2rx* genes. Representative embryos from stages (St.) 22/23–41 displaying *p2rx* subunit expression detected by whole-mount *in situ* hybridization. To further characterize their expression profile, embryos stained for *p2rx* genes, except *p2r6.L* and *p2rx7.L*, were selected for scalpel sections and cryostat sections. Sections were performed on stage 22/23 for *p2rx5* stained embryos, on stage 33/34 for *p2rx1.L* stained embryos, on stage 37/38 for *p2rx1.S*, *p2rx2.S*, and *p2rx4.L* stained embryos and stage 41 for *p2rx2.L* stained embryos. The red numbered dotted arrows indicate the plan of the sections. For whole mount pictures, dorsal is up and anterior is right except for the *p2rx5 and p2rx7* subunits for which a dorsal view completes the lateral view. For section pictures, dorsal is up. The green arrowhead indicates the fused ganglion of middle lateral line and glossopharyngeal nerves, the orange one shows the trigeminal ganglion and ganglion of anterodorsal lateral line nerve and the red one the ganglia of anteroventral lateral line and facial nerves. c.g, cement gland; e, eye; fb, forebrain; g.b, gall bladder; g.l, ganglion layer; h, heart; hb, hindbrain; l, lens; mb, midbrain; n, notochord; o.n.l, outer nuclear layer; o.v, otic vesicle; p.g, pineal gland; p.gl, pronephric glomus; p.t, pronephric tubules; s, somite; t.n, trigeminal nerve; v.b.i, ventral blood island.

The spatial expression of these genes in the embryo was assessed by whole-mount *in situ* hybridization, performed from stage 5 to stage 41 with specific antisense riboprobes (Figure 3). Probes were designed in order to discriminate the expression of the homeologs except for *p2rx5* genes. Probes were designed in the 3′UTR region except for *p2rx5*, *p2rx6*, *p2rx7* for which genes probes are located in the coding region. For each subunit, controls were carried out with sense probes to discriminate specific and unspecific signals (see Figure 4). Sectioning of representative p2rx stained embryos was performed in order to further characterize their expression profile (Figure 5). As shown in Figures 3, 5, p2rx subunits display specific and different, but also overlapping expression domains during *X. laevis* development. Expression profile of the different *p2rx* subunits is summarized in Table 2.

**TABLE 2:** Summary of *p2rx* subunit expression domains in *Xenopus* late organogenesis stage embryos.

**P2rx mRNA**	**1.L**	**1.S**	**2.L**	**2.S**	**4.L**	**5**	**6.L**	**7.L**
**Neural structures**								
CNS Forebrain Midbrain Hindbrain Pineal gland Trigeminal nerve	+ + +	+ + +	+ + + + + + + +		+ + +			+ +
Eye Retina		+	+ +					+
Otic Vesicle			+					+
Sensory ganglia ad.l.l. /t.g av.l.l./f.n m.l.l/g.n		+ + + + + +	+ + + + + +				+ + +	
**Non neural structures**								
Branchial arches	+							
Cement gland	+ +				+			
Gall bladder			+ +					
Eye Lens		+	+ +	+				
Kidney Glomus Tubules (P.T)				+ + +	+ + + +			
Heart	+ + +		+					
Ventral blood island			+ + +					
Vascular system	+ +		+ +					
Somites			+			+ + + +	+	

*Expression levels are indicated on a semi quantitative scale. The number of crosses (+) indicates relative expression levels. ad.l.l./t.g, ganglia of anterodorsal lateral line and trigeminal nerves; av.l.l./f.n, ganglia of anteroventral lateral line and facial nerves; m.l.l/g.n, Fused ganglion of middle lateral line and glossopharyngeal nerves; CNS, Central Nervous System; System; P.T, Proximal Tubules.*

*P2rx1* homeologs display distinct expression profile. The *p2rx1.L* subunit is not detected before stage 27 (Figure 3). At this stage and stage 33/34, its transcripts are localized in a very specific region of the hindbrain, in a two rows of laterally symmetrical neurons, in a ventral position along the dorso-ventral axis (Figure 5). At stage 33/34, *p2rx1.L* expression is also detected in the head region, in the branchial arches and in the cement gland, a mucus-secreting ectodermal structure. At later stages, staining is also found in the heart and in the vascular network between the heart and kidney (Figure 3). However, its expression in the notochord is unspecific as detected with the sense probe (Figure 4). Expression of the *p2rx1.S* homeolog is detected from cleavage stage (Figure 3). At stage 5, its expression is observed in some blastomeres at the animal pole. No specific signal was detected during gastrulation. During neurulation, its transcripts are detected in the developing nervous system and in the presumptive eye field. Its expression remains in the developing eye until stage 41. Transverse sections show expression in the retina layers (Figure 5). From stage 27/28, weak diffuse expression is observed in the head and dorsal region of the embryo. At later stages, strong *p2rx1.S* expression is observed into two specific domains of the neurogenic placodes and peripheral nervous system: in the fused ganglion of middle lateral line and glossopharyngeal nerves as well as in the trigeminal ganglion and ganglion of anterodorsal lateral line nerve (Figure 5).

The homeologs *p2rx2* also display different expression pattern. *P2rx2.L* transcripts are only detected in specific manner from early organogenesis stages, the staining during segmentation phase being observed with the sense probe (Figure 3). During organogenesis, *p2rx2.L* expression is mostly detected in developing mesoderm derivatives and nervous system. At early tailbud phase (st. 22/23), weak staining can be observed in the somites. At tailbud stages, staining is found in the ventral blood island from stage 27, in the duct of Cuvier from stage 33/34 and in the heart from stage 37 (Figures 3, 5). At stage 41, expression can be observed in the gall bladder (Figure 5). Transcripts are also found in the head region, in the brain and the sensory organs such as eye and otic vesicule. At stage 41, staining in the eye is detected both in the retina, in the retinal ganglion and outer nuclear layers, and in the lens (Figure 5). From stage 37/38, *p2rx2.L* transcripts are detected in the trigeminal nerve (ophthalmic and maxillary branches) and strong expression is detected into specific regions of neurogenic placodes and peripheral nervous system : in the fused ganglion of middle lateral line and glossopharyngeal nerves and in ganglia of anteroventral lateral line and facial nerves (Figures 3, 5). The *p2rx2.S* subunit displays a very tissue specific expression domain, with undetectable neural expression (Figure 5). No expression can be detected before stage 37 (Figure 3). From stage 37–41, its expression is only detected in the kidney region, in the pronephric glomus, as signal in the head and notochord is unspecific (Figures 3–5).

The *p2rx4.L* subunit also displays a very specific expression domain. As expected from the RT-PCR results, strong staining is observed in the animal pole in blastula and in the involuting mesoderm in gastrula embryos (Figure 3). However, no zygotic expression is detected before stage 33/34. At this stage, its transcripts are mostly found in the kidney. At stage 37/38, *p2rx4.L* is highly expressed in the proximal tubules (Figure 5). Expression is also observed in the pineal gland (epiphysis) (Figure 3).

The staining domain observed with the common *p2rx5* riboprobe, which does not discriminate the two homeologs, is also very specific, only in mesodermal and not neural tissues (Figure 3). From stage 22/23, *p2rx5* genes are only and highly expressed in the developing somites (Figures 3–5). This restricted muscle expression is unique to these members of the family.

The *p2rx6.L* and *p2rx7.L* genes are more weakly expressed in the embryo (Figure 3). At stage 33/34, weak *p2rx6.L* expression is observed in the somites. At stage 41, strong expression is detected in the head region and dorsal region of the embryo but this staining is unspecific as observed with the sense probe (Figure 4). However, the expression observed in the fused ganglion of middle lateral line and glossopharyngeal nerves is specific (Figure 5). The *p2rx7.L* subunit is only weakly but specifically detected at stage 41, in the head region, in the forebrain and midbrain and sensory organs (eye and otic vesicule) (see Figures 4, 5).

## Discussion

We here report the cloning of the complete *p2rx* gene family in *Xenopus* in addition to p2rx4 and p2rx7 members previously identified (Juranka et al., 2001; Paukert et al., 2002). Moreover, we provide a complete expression map of all *p2rx* genes throughout *X. laevis* embryonic life. This is the first study to compare the expression of the entire p2rx family during the early phases of development, notably the earliest ones such as gastrulation and neurulation, corresponding to the specification and formation of the neural tube.

### Evolutionary Conserved p2rx Subunit Genes Are Present in *Xenopus*, Except p2rx3

Our study reveals that all members of the *p2rx* family, except *p2rx3*, are present in *Xenopus laevis* and *tropicalis*. Phylogenetic analysis demonstrates the high degree of conservation during vertebrate evolution both at the protein and genomic level. The two transmembrane domains positions are conserved in all amphibian P2X subunits and the exons/introns boundaries are also conserved except for the boundary 11 (separating exons 11 and 12), which is only conserved between homeologs and orthologs. The full length of cDNAs, proteins and genomic sequences of all P2X subunits was used for our phylogenetic analysis. C-terminus tail sequences, encoded by exon 12 or exons 12 and 13 for p2rx7.L, were retained in our analysis although they correspond to the most variable domain in terms in length and sequences. The percentage of identity between the different *X. laevis* P2X subunits is similar to those shared by mammalian ones (North, 2002), the most conserved region being the transmembrane domains and the extracellular loop. Sixty four amino acids (AA) are conserved in all 15 *Xenopus* p2rx sequences, with 56/64 being located in the extracellular domain sequence. In the intracellular N-terminal domain, only a motif of 3 amino acids (Thr-X-Arg/Lys, with X being any amino acid) is conserved. This motif is a phosphorylation site by the Protein Kinase C modulating the desensitization rate of mammalian P2X receptors (Boué-Grabot et al., 2000b). In the intracellular C-terminal domain, the consensus motif Tyr-XXX-Lys (with X being any amino acid) involved in surface targeting of mammalian P2X receptors is also conserved in all *Xenopus* P2X subunits (Chaumont et al., 2004). In the extracellular domain, the 10 cysteines (located at position 113, 124, 130, 147, 158, 164, 214, 224, 258, 267 in rat P2X2 and indicated with an asterisk on the Supplementary Figure S1) involved in tertiary structure of P2X subunit by disulphide bonds formation are conserved in *Xenopus* (North, 2002). Furthermore, 7 of the 8 AA involved in ATP binding are also conserved [Lys70, Lys72, Phe188, Thr189, Asn296, Phe297, Arg298, Lys316 in zebrafish P2X4 (Habermacher et al., 2016); indicated with an black triangle on Supplementary Figure S1]. The Phe297 amino acid (indicated with an open triangle on Supplementary Figure S1) is conserved in all, except P2X2, *Xenopus* sequences. This is quite surprising as this phenylalanine is present in the mammalian P2X2 orthologous sequences.

Intriguingly, no genomic sequence for *p2rx3* was identified suggesting the loss of this subunit in *Pipidae*. Search on Ensembl and NCBI databases shows that this subunit is present in the different classes of vertebrates [mammalia, reptilia, aves, fish (cartilaginous, ray-finned fishes and coelacanths)] but not in agnatha. In vertebrates, P2X3 receptor is predominantly expressed in nociceptor sensory neurons of dorsal root and trigeminal ganglia and plays a key role in pain transduction (Vulchanova et al., 1997; Xiang et al., 1998; Boué-Grabot et al., 2000a; Norton et al., 2000; Kucenas et al., 2003; Appelbaum et al., 2007). In zebrafish or pufferfish, P2X3 is specifically expressed in trigeminal neurons and spinal cord Rohon-Beard cells and functional studies using transgenic animals showed its implication in sensory circuit formation during development (Kucenas et al., 2006, 2009; Palanca et al., 2013). In rodent P2X3 is similarly expressed in trigeminal and dorsal root ganglia sensory neurons and P2X3KO mice demonstrated its implication in nociception as well as sensory transduction (Cockayne et al., 2000; Souslova et al., 2000). Zebrafish p2rx3.2 is co-expressed with the ectonucleotidase entpdase3 in spinal cord Rohon-Beard cells (Appelbaum et al., 2007). We previously published entpdase3 expression in *Xenopus* Rohon-Beard cells (Massé et al., 2006), making amphibian *p2rx3* gene absence even more unexpected. Our *in situ* hybridization data show that *p2rx1.S*, *p2rx2.L*, and *p2rx6.L* are expressed in cranial neurogenic placodes, in the trigeminal ganglion and sensory lateral line system. Future work will have to address if one of these subunits could fulfill the functions of P2X3.

### The Expression Patterns of *p2rx* Genes Are Temporally and Spatially Regulated

*Xenopus laevis p2rx* genes are all expressed during embryogenesis although their expression patterns differ during frog development. Only two subunits, *p2rx4.L* and *p2rx6.L*, are maternally expressed. *P2rx4* displays the highest level of expression in egg and early embryo stages (before the MBT), which is in agreement with its expression in oocytes (Juranka et al., 2001). Based on their zygotic expression, *p2rx* subunits can be divided into two groups: those which are not expressed in the nervous system, *p2rx2.S and p2rx5*, and those which are expressed in the nervous system, although their expression can not be restricted to this tissue. For example, *p2rx4.L*, expressed in the pineal gland, is also highly expressed in the pronephros, *p2rx2.L* transcripts, found in sensory system, are also detected in the cardiovascular system or *p2rx1.L* expression detected at high level in an interneuron population in the hindbrain is also found in the cement gland. These distinct expression profiles suggest specific functions for these receptors during embryogenesis, during the formation of neural and non-neural tissues. However, as in mouse and zebrafish embryos, *p2rx7.L* does not seem to be involved during early embryogenesis as its expression is only detected weakly at late organogenesis stages (Kucenas et al., 2003; Appelbaum et al., 2007; MGI website). The absence of gross phenotype at birth of null P2X7 mice is in agreement with this result (Solle et al., 2001), although its activity has been reported, *in vitro*, as essential for mouse embryonic stem cells survival (Thompson et al., 2012).

### Conserved Expression Domains in Mesodermic Derivatives

Several *p2rx subunits* display specific and almost restricted expression profile in two mesodermic derivatives, the kidney and somites. These expression patterns have been conserved during evolution. Our data demonstrate that *p2xr5* subunit is highly expressed in the somitic tissues. In rat and chick embryos, P2X5 is the earliest P2X receptors expressed in somites (Meyer et al., 1999; Ruppelt et al., 2001; Ryten et al., 2001). Somitic expression of this subunit has also been reported in zebrafish (Kucenas et al., 2003). *In vitro* studies demonstrated that P2X5 is involved in myogenesis and muscle progenitor cells (satellite cells) differentiation (Ryten et al., 2002; Araya et al., 2004; Martinello et al., 2011). Its possible implication during muscle regeneration makes this receptor a therapeutic target for musculoskeletal pathologies (Ryten et al., 2004; Burnstock et al., 2013). In rat, P2X2 and P2X6 are also expressed in developing skeletal muscles (Ryten et al., 2001). We observed *p2rx2.L and p2xr6.L* expression in *Xenopus* somites, although their transcripts are only detected at a low level compared to *p2rx5* somitic expression level. This suggests that P2X5 may be the major P2 receptor regulating somitogenesis. The easiness of *Xenopus* as an *in vivo* model for myogenesis makes this vertebrate embryo an ideal model to apprehend the functional roles of P2X5 during myogenesis (Sabillo et al., 2016).

*P2rx4.L and p2rx2.S* are both expressed at high level in the embryonic functional kidney, the pronephros, but in different regions: p2rx2.S is expressed in the glomus, the filtration unit, whereas *p2rx4* is expressed in the proximal tubules. No *p2rx* transcripts were detected in the distal pronephric region, e.g., the distal, and connecting tubules. In the rat metanephros, P2X2 is found in the glomerulus and collecting duct where it may be involved in ATP inhibitory effects on AVP-induced water permeability (Burnstock et al., 2014). In mammalian metanephros P2RX4 is one of the major P2X receptors and its expression is found along the entire nephron (glomerulus, proximal tubules, descending and ascending limb of Henle, distal tubules, and collecting duct) (reviewed in Bailey et al., 2000; Burnstock et al., 2014; Menzies et al., 2017). P2X4 and P2X4/6 receptors are involved in diverse physiological renal processes, depending on its nephron localization and but also in renal pathology, such as diabetic nephropathy (Chen et al., 2013; Menzies et al., 2017). Several evidence suggest that P2X4 may form complexes with P2X7 in the metanephros (Craigie et al., 2013) but we failed to detect any *p2rx6* or *p2xr7* expression in the developing amphibian pronephros, suggesting no renal hetero-oligomerization between P2X4 and these receptors in *Xenopus*. As in mammals, the *Xenopus* kidney is one of the major sites of expression of the purinergic receptors and ectonucleotidases. We previously published the renal expression of the ectonucleotidases entpdase 5 and enpp2, enpp4, enpp6 (Massé et al., 2010, 2006). Due to its simple structural organization, composed of a single enormous nephron, *Xenopus* pronephros may be an ideal model to address the functions of the P2X receptors and the purinergic signaling during kidney formation, renal physiology but also pathophysiology (Lienkamp, 2016; Krneta-Stankic et al., 2017).

### Function During the Development of Central and Peripheral Nervous System

The developing nervous system is the major site of expression of the *p2rx* genes, with 7 out of the 9 genes displaying an expression in the central or peripheral nervous system. Surprisingly, only the expression of *p2rx1.S* was detected by *in situ* hybridization at stage neurula in developing nervous system, although *p2rx2.L, p2rx2.S* transcripts were amplified during neurulation by RT-PCR, a more sensitive technique, suggesting they may also be involved in neural tube formation. Little information is available regarding *p2rx* expression at neurula stages in other vertebrate models, although P2X3 seems to be the earliest subunit expressed during central nervous system formation (Massé and Dale, 2012; Burnstock and Dale, 2015). Developing central nervous system is a major site of expression of the *p2rx* genes, with 5 out of the 9 expressed in this tissue displaying diffuse and/or weak expresssion, for example *p2rx7.L*, or specific and strong expression. Such discrete domains are the pineal gland that expresses *p2rx4.L* or the subset of hindbrain neurons labeled with the *p2rx1.L* antisense probe. The ventral localization of these neurons suggests they might be a subset of interneurons or motor neurons (Thélie et al., 2015).

Several *p2xr* subunits are expressed in the cranial sensory peripheral nervous system at later organogenesis stages after the neurogenic placode differentiation (Schlosser and Northcutt, 2000). P2rx2.L is expressed in trigeminal ganglia. *p2rx1.S, p2rx2.S*, and *p2rx6.L are* expressed in *Xenopus* ganglia of the anterodorsal, anteroventral and middle lateral line nerves, which are mechanosensory organs, composed of mechanoreceptive neuromasts involved in the detection of water displacements and current and electric fields (Shelton, 1971; Winklbauer, 1989). Although, p2xr3, the prototypic sensory P2X subtypes, is absent in *Xenopus laevis*, our data suggest that P2X receptors might have functional implication in sensory transmission rather than in neurogenic placode differentiation (Nakatsuka and Gu, 2006). *p2rx1.S, p2rx2.S*, and *p2rx6.L* mRNAs are colocalized into the ganglia of glossopharyngeal and middle lateral line nerves, suggesting co-expression of these subunits. Several biochemical evidences suggested the existence of P2X1/2 complexes, but still not confirmed *in vivo* (reviewed in Saul et al., 2013). However, existence of functional P2X2/6 channels and the co-expression of these two subunits in several rat neuronal populations have been demonstrated (Collo et al., 1996; King et al., 2000). Future work will need to address *in vivo* the potential oligo-heteromerization of these subunits during vertebrate neurogenesis.

Taken together, this work is a fundamental prerequisite to apprehend the developmental functions of P2X receptors during vertebrate embryogenesis. Specific gain or loss of function studies of the P2X subunits identified in this work should allow to decipher their functions during the early and late phases of the formation of the central and peripheral nervous system.

## Data Availability

The datasets generated for this study can be found in Genbank, ID 2203640.

## Ethics Statement

This study was carried out in strict accordance with the recommendations in the Guide for the Care and Use of Laboratory Animals of the European Community and approved by the ethical committee of Bordeaux.

## Author Contributions

CB performed the experiments, analyzed the data, and corrected the manuscript. EB-G made the figures and wrote the manuscript. KM supervised the work, analyzed the data, and wrote the manuscript.

## Conflict of Interest Statement

The authors declare that the research was conducted in the absence of any commercial or financial relationships that could be construed as a potential conflict of interest.

## Footnotes

1 http://web.expasy.org/translate/

## References

[B1] Altschul S. F. Gish W. Miller W. Myers E. W. Lipman D. J. (1990). Basic local alignment search tool. *J. Mol. Biol.* 215 403–410. 10.1016/S0022-2836(05)80360-2 2231712

[B2] Appelbaum L. Skariah G. Mourrain P. Mignot E. (2007). Comparative expression of p2x receptors and ecto-nucleoside triphosphate diphosphohydrolase 3 in hypocretin and sensory neurons in zebrafish. *Brain Res.* 1174 66–75. 10.1016/j.brainres.2007.06.103 17868657

[B3] Araya R. Riquelme M. A. Brandan E. Sáez J. C. (2004). The formation of skeletal muscle myotubes requires functional membrane receptors activated by extracellular ATP. *Brain Res. Brain Res. Rev.* 47 174–188. 10.1016/j.brainresrev.2004.06.003 15572171

[B4] Artimo P. Jonnalagedda M. Arnold K. Baratin D. Csardi G. de Castro E. (2012). ExPASy: sIB bioinformatics resource portal. *Nucleic Acids Res.* 40 W597–W603. 10.1093/nar/gks400 22661580 PMC3394269

[B5] Bailey M. A. Hillman K. A. Unwin R. J. (2000). P2 receptors in the kidney. *J. Auton. Nerv. Syst.* 81 264–270. 10.1016/s0165-1838(00)00125-9 10869730

[B6] Bassez T. Paris J. Omilli F. Dorel C. Osborne H. B. (1990). Post-transcriptional regulation of ornithine decarboxylase in *Xenopus laevis* oocytes. *Development* 110 955–962. 10.1242/dev.110.3.955 2088731

[B7] Blanchard C. Massé K. (2019). “Developmental expression of ectonucleotidase and purinergic receptors detection by whole-mount *in situ* hybridization in embryos in the methods molecular biology,” in *Purinergic Signaling.* Springer: Nature 10.1007/978-1-4939-9717-6_6 31646482

[B8] Boué-Grabot E. Akimenko M. A. Séguéla P. (2000a). Unique functional properties of a sensory neuronal P2X ATP-gated channel from zebrafish. *J. Neurochem.* 75 1600–1607. 10.1074/jbc.275.14.10190 10987841

[B9] Boué-Grabot E. Archambault V. Séguéla P. (2000b). A protein kinase C site highly conserved in P2X subunits controls the desensitization kinetics of P2X2 ATP-gated channels. *J. Biol. Chem.* 275 10190–10195. 10.1074/jbc.275.14.10190 10744703

[B10] Boué-Grabot E. Pankratov Y. (2017). Modulation of central synapses by astrocyte-released atp and postsynaptic P2X receptors. *Neural Plast.* 2017:9454275. 10.1155/2017/9454275 28845311 PMC5563405

[B11] Breathnach R. Chambon P. (1981). Organization and expression of eucaryotic split genes coding for proteins. *Annu. Rev. Biochem.* 50 349–383. 10.1146/annurev.bi.50.070181.002025 6791577

[B12] Burnstock G. (2014). Purinergic signalling: from discovery to current developments. *Exp. Physiol.* 99 16–34. 10.1113/expphysiol.2013.071951 24078669 PMC4208685

[B13] Burnstock G. (2016). Purinergic mechanisms and pain. *Adv. Pharmacol.* 75 91–137. 10.1016/bs.apha.2015.09.001 26920010

[B14] Burnstock G. (2017). Purinergic signalling and neurological diseases: an update. *CNS Neurol. Disord. Drug Targets* 16 257–265. 10.2174/1871527315666160922104848 27658510

[B15] Burnstock G. (2018). The therapeutic potential of purinergic signalling. *Biochem. Pharmacol.* 151 157–165. 10.1016/j.bcp.2017.07.016 28735873

[B16] Burnstock G. Arnett T. R. Orriss I. R. (2013). Purinergic signalling in the musculoskeletal system. *Purinergic Signal.* 9 541–572. 10.1007/s11302-013-9381-4 23943493 PMC3889393

[B17] Burnstock G. Dale N. (2015). Purinergic signalling during development and ageing. *Purinergic Signal.* 11 277–305. 10.1007/s11302-015-9452-9 25989750 PMC4529855

[B18] Burnstock G. Evans L. C. Bailey M. A. (2014). Purinergic signalling in the kidney in health and disease. *Purinergic Signal.* 10 71–101. 10.1007/s11302-013-9400-5 24265071 PMC3944043

[B19] Burnstock G. Krügel U. Abbracchio M. P. Illes P. (2011). Purinergic signalling: from normal behaviour to pathological brain function. *Prog. Neurobiol.* 95 229–274. 10.1016/j.pneurobio.2011.08.006 21907261

[B20] Chaumont S. Jiang L. H. Penna A. North R. A. Rassendren F. (2004). Identification of a trafficking motif involved in the stabilization and polarization of P2X receptors.: trafficking motif of P2X ATP-gated channels. *J. Biol. Chem.* 279 29628–29638. 10.1074/jbc.M403940200 15126501

[B21] Chen K. Zhang J. Zhang W. Zhang J. Yang J. Li K. (2013). ATP-P2X4 signaling mediates NLRP3 inflammasome activation: a novel pathway of diabetic nephropathy. *Int. J. Biochem. Cell Biol.* 45 932–943. 10.1016/j.biocel.2013.02.009 23434541

[B22] Cockayne D. A. Hamilton S. G. Zhu Q. M. Dunn P. M. Zhong Y. Novakovic S. (2000). Urinary bladder hyporeflexia and reduced pain-related behaviour in P2X3-deficient mice. *Nature* 407 1011–1015. 10.1038/35039519 11069181

[B23] Collo G. North R. A. Kawashima E. Merlo-Pich E. Neidhart S. Surprenant A. (1996). Cloning OF P2X5 and P2X6 receptors and the distribution and properties of an extended family of ATP-gated ion channels. *J. Neurosci.* 16 2495–2507. 10.1523/jneurosci.16-08-02495.1996 8786426 PMC6578782

[B24] Craigie E. Birch R. E. Unwin R. J. Wildman S. S. (2013). The relationship between P2X4 and P2X7: a physiologically important interaction? *Front. Physiol.* 4:216. 10.3389/fphys.2013.00216 23966951 PMC3744038

[B25] Del Puerto A. Wandosell F. Garrido J. J. (2013). Neuronal and glial purinergic receptors functions in neuron development and brain disease. *Front. Cell Neurosci.* 7:197 10.3389/fncel.2013.00197 PMC3808753 24191147

[B26] Dereeper A. Guignon V. Blanc G. Audic S. Buffet S. Chevenet F. (2008). Phylogeny.fr: robust phylogenetic analysis for the non-specialist. *Nucleic Acids Res.* 36 W465–W469. 10.1093/nar/gkn180 18424797 PMC2447785

[B27] Domercq M. Zabala A. Matute C. (2018). Purinergic receptors in multiple sclerosis pathogenesis. *Brain Res. Bull.* 10.1016/j.brainresbull.2018.11.018 [Epub ahead of print]. 30500565

[B28] Fumagalli M. Lecca D. Abbracchio M. P. Ceruti S. (2017). Pathophysiological role of purines and pyrimidines in neurodevelopment: unveiling new pharmacological approaches to congenital brain diseases. *Front. Pharmacol.* 8:941. 10.3389/fphar.2017.00941 29375373 PMC5770749

[B29] Habermacher C. Dunning K. Chataigneau T. Grutter T. (2016). Molecular structure and function of P2X receptors. *Neuropharmacology* 104 18–30. 10.1016/j.neuropharm.2015.07.032 26231831

[B30] Hellsten U. Harland R. M. Gilchrist M. J. Hendrix D. Jurka J. Kapitonov V. (2010). The genome of the Western clawed frog *Xenopus tropicalis*. *Science* 328 633–636. 10.1126/science.1183670 20431018 PMC2994648

[B31] Juranka P. F. Haghighi A. P. Gaertner T. Cooper E. Morris C. E. (2001). Molecular cloning and functional expression of *Xenopus laevis* oocyte ATP-activated P2X4 channels. *Biochim. Biophys. Acta* 1512 111–124. 10.1016/s0005-2736(01)00313-3 11334629

[B32] Kawate T. Michel J. C. Birdsong W. T. Gouaux E. (2009). Crystal structure of the ATP-gated P2X(4) ion channel in the closed state. *Nature* 460 592–598. 10.1038/nature08198 19641588 PMC2720809

[B33] Khakh B. S. North R. A. (2006). P2X receptors as cell-surface ATP sensors in health and disease. *Nature* 442 527–532. 10.1038/nature04886 16885977

[B34] Khakh B. S. North R. A. (2012). Neuromodulation by extracellular ATP and P2X receptors in the CNS. *Neuron* 76 51–69. 10.1016/j.neuron.2012.09.024 23040806 PMC4064466

[B35] King B. F. Townsend-Nicholson A. Wildman S. S. Thomas T. Spyer K. M. Burnstock G. (2000). Coexpression of rat P2X2 and P2X6 subunits in *Xenopus* oocytes. *J. Neurosci.* 20 4871–4877. 10.1523/JNEUROSCI.20-13-04871.2000 PMC6772291 10864944

[B36] Krneta-Stankic V. DeLay B. D. Miller R. K. (2017). *Xenopus*: leaping forward in kidney organogenesis. *Pediatr. Nephrol.* 32 547–555. 10.1007/s00467-016-3372-y 27099217 PMC5074909

[B37] Kucenas S. Cox J. A. Soto F. Lamora A. Voigt M. M. (2009). Ectodermal P2X receptor function plays a pivotal role in craniofacial development of the zebrafish. *Purinergic Signal.* 5 395–407. 10.1007/s11302-009-9165-z 19529983 PMC2717322

[B38] Kucenas S. Li Z. Cox J. A. Egan T. M. Voigt M. M. (2003). Molecular characterization of the zebrafish P2X receptor subunit gene family. *Neuroscience.* 121 935–945. 10.1016/s0306-4522(03)00566-9 14580944

[B39] Kucenas S. Soto F. Cox J. A. Voigt M. M. (2006). Selective labeling of central and peripheral sensory neurons in the developing zebrafish using P2X_3_ receptor subunit transgenes. *Neuroscience* 138 641–652. 10.1016/j.neuroscience.2005.11.058 16413125

[B40] Lienkamp S. S. (2016). Using *Xenopus* to study genetic kidney diseases. *Semin. Cell Dev. Biol.* 51 117–124. 10.1016/j.semcdb.2016.02.002 26851624

[B41] Martinello T. Baldoin M. C. Morbiato L. Paganin M. Tarricone E. Schiavo G. (2011). Extracellular ATP signaling during differentiation of C2C12 skeletal muscle cells: role in proliferation. *Mol. Cell. Biochem.* 351 183–196. 10.1007/s11010-011-0726-4 21308481

[B42] Massé K. Bhamra S. Allsop G. Dale N. Jones E. A. (2010). Ectophosphodiesterase/nucleotide phosphohydrolase (Enpp) nucleotidases: cloning, conservation and developmental restriction. *Int. J. Dev. Biol.* 54 181–193. 10.1387/ijdb.092879km 19598106

[B43] Massé K. Bhamra S. Eason R. Dale N. Jones E. A. (2007). Purine-mediated signalling triggers eye development. *Nature* 449 1058–1062. 10.1038/nature06189 17960245

[B44] Massé K. Dale N. (2012). Purines as potential morphogens during embryonic development. *Purinergic Signal.* 8 503–521. 10.1007/s11302-012-9290-y 22270538 PMC3360092

[B45] Massé K. Eason R. Bhamra S. Dale N. Jones E. A. (2006). Comparative genomic and expression analysis of the conserved NTPDase gene family in *Xenopus*. *Genomics* 87 366–381. 10.1016/j.ygeno.2005.11.003 16380227

[B46] Menzies R. I. Tam F. W. Unwin R. J. Bailey M. A. (2017). Purinergic signaling in kidney disease. *Kidney Int.* 91 315–323. 10.1016/j.kint.2016.08.029 27780585

[B47] Meyer M. P. Gröschel-Stewart U. Robson T. Burnstock G. (1999). Expression of two ATP-gated ion channels, P2X_5_ and P2X_6_, in developing chick skeletal muscle. *Dev. Dyn.* 216 442–449. 10.1002/(SICI)1097-0177(199912)216:4/5<442::AID-DVDY12>3.0.CO;2-Z 10633863

[B48] Nakatsuka T. Gu J. G. (2006). P2X purinoceptors and sensory transmission. *Pflugers Arch.* 452 598–607. 10.1007/s00424-006-0057-6 16547751

[B49] Needleman S. B. Wunsch C. D. (1970). A general method applicable to the search for similarities in the amino acid sequence of two proteins. *J. Mol. Biol.* 48 443–453. 10.1016/0022-2836(70)90057-4 5420325

[B50] Nieuwkoop P. D. Faber J. (1994). *Normal Table of Xenopus Laevis (Daudin).* New York, NY: Garland Publishing, Inc.

[B51] North R. A. (2002). Molecular physiology of P2X receptors. *Physiol. Rev.* 82 1013–1067. 10.1152/physrev.00015.2002 12270951

[B52] North R. A. (2016). P2X receptors. *Philos. Trans. R. Soc. Lond. B Biol. Sci.* 371:20150427. 10.1098/rstb.2015.0427 27377721 PMC4938027

[B53] Norton W. H. Rohr K. B. Burnstock G. (2000). Embryonic expression of a P2X(3) receptor encoding gene in zebrafish. *Mech. Dev.* 99 149–152. 10.1016/s0925-4773(00)00472-x 11091083

[B54] Oliveira Á Illes P. Ulrich H. (2016). Purinergic receptors in embryonic and adult neurogenesis. *Neuropharmacology* 104 272–281. 10.1016/j.neuropharm.2015.10.008 26456352

[B55] Palanca A. M. Lee S. L. Yee L. E. Joe-Wong C. Trinh le A. Hiroyasu E. (2013). New transgenic reporters identify somatosensory neuron subtypes in larval zebrafish. *Dev. Neurobiol* 73 152–167. 10.1002/dneu.22049 22865660 PMC3541445

[B56] Paukert M. Hidayat S. Gründer S. (2002). The P2X(7) receptor from *Xenopus laevis*: formation of a large pore in *Xenopus* oocytes. *FEBS Lett.* 513 253–258. 10.1016/s0014-5793(02)02324-4 11904160

[B57] Ralevic V. Burnstock G. (1998). Receptors for purines and pyrimidines. *Pharmacol. Rev.* 50 413–492. 9755289

[B58] Rodrigues R. J. Marques J. M. Cunha R. A. (2018). Purinergic signalling and brain development. *Semin. Cell Dev. Biol.* 10.1016/j.semcdb.2018.12.001 [Epub ahead of print]. 30529149

[B59] Ruppelt A. Ma W. Borchardt K. Silberberg S. D. Soto F. (2001). Genomic structure, developmental distribution and functional properties of the chicken P2X_5_ receptor. *J. Neurochem.* 77 1256–1265. 10.1046/j.1471-4159.2001.00348.x 11389176

[B60] Ryten M. Dunn P. M. Neary J. T. Burnstock G. (2002). ATP regulates the differentiation of mammalian skeletal muscle by activation of a P2X5 receptor on satellite cells. *J. Cell Biol.* 158 345–355. 10.1083/jcb.200202025 12135987 PMC2173112

[B61] Ryten M. Hoebertz A. Burnstock G. (2001). Sequential expression of three receptor subtypes for extracellular ATP in developing rat skeletal muscle. *Dev. Dyn.* 221 331–341. 10.1002/dvdy.1147 11458393

[B62] Ryten M. Yang S. Y. Dunn P. M. Goldspink G. Burnstock G. (2004). Purinoceptor expression in regenerating skeletal muscle in the mdx mouse model of muscular dystrophy and in satellite cell cultures. *FASEB J.* 18 1404–1406. 10.1096/fj.03-1175fje 15231720

[B63] Sabillo A. Ramirez J. Domingo C. R. (2016). Making muscle: morphogenetic movements and molecular mechanisms of myogenesis in *Xenopus laevis*. *Semin. Cell Dev. Biol .* 51 80–91. 10.1016/j.semcdb.2016.02.006 26853935 PMC4798873

[B64] Sater A. K. Moody S. A. (2017). Using *Xenopus* to understand human disease and developmental disorders. *Genesis* 55:e22997. 10.1002/dvg.22997 28095616

[B65] Saul A. Hausmann R. Kless A. Nicke A. (2013). Heteromeric assembly of P2X subunits. *Front. Cell. Neurosci.* 7:250. 10.3389/fncel.2013.00250 24391538 PMC3866589

[B66] Schlosser G. Northcutt R. G. (2000). Development of neurogenic placodes in *Xenopus laevis*. *J. Comp. Neurol.* 418 121–146. 10.1002/(SICI)1096-9861(20000306)418:2<121::AID-CNE1>3.0.CO;2-M 10701439

[B67] Session A. M. Uno Y. Kwon T. Chapman J. A. Toyoda A. Takahashi S. (2016). Genome evolution in the allotetraploid frog *Xenopus laevis*. *Nature* 538 336–343. 10.1038/nature19840 27762356 PMC5313049

[B68] Shelton P. M. (1971). The structure and function of the lateral line system in larval *Xenopus laevis*. *J. Exp. Zool.* 178 211–231. 10.1002/jez.1401780207 5114041

[B69] Solle M. Labasi J. Perregaux D. G. Stam E. Petrushova N. Koller B. H. (2001). Altered cytokine production in mice lacking P2X(7) receptors. *J. Biol. Chem.* 276 125–132. 10.1074/jbc.M006781200 11016935

[B70] Souslova V. Cesare P. Ding Y. Akopian A. N. Stanfa L. Suzuki R. (2000). Warm-coding deficits and aberrant inflammatory pain in mice lacking P2X3 receptors. *Nature* 407 1015–1017. 10.1038/35039526 11069182

[B71] Tandon P. Conlon F. Furlow J. D. Horb M. E. (2017). Expanding the genetic toolkit in *Xenopus*: approaches and opportunities for human disease modeling. *Dev. Biol.* 426 325–335. 10.1016/j.ydbio.2016.04.009 27109192 PMC5074924

[B72] Thélie A. Desiderio S. Hanotel J. Quigley I. Van Driessche B. Rodari A. (2015). Prdm12 specifies V1 interneurons through cross-repressive interactions with Dbx1 and Nkx6 genes in *Xenopus*. *Development* 142 3416–3428. 10.1242/dev.121871 26443638 PMC4631751

[B73] Thompson B. A. Storm M. P. Hewinson J. Hogg S. Welham M. J. MacKenzie A. B. (2012). A novel role for P2X7 receptor signalling in the survival of mouse embryonic stem cells. *Cell. Signal.* 24 770–778. 10.1016/j.cellsig.2011.11.012 22120528 PMC3271386

[B74] Thompson J. D. Higgins D. G. Gibson T. J. (1994). CLUSTAL W: improving the sensitivity of progressive multiple sequence alignment through sequences weighting, position-specific gap penalties and weight matrix choice. *Nucleic Acids Res.* 22 4673–4680. 10.1093/nar/22.22.4673 7984417 PMC308517

[B75] Tocco A. Pinson B. Thiébaud P. Thézé N. Massé K. (2015). Comparative genomic and expression analysis of the adenosine signaling pathway members in *Xenopus*. *Purinergic Signal.* 11 59–77. 10.1007/s11302-014-9431-6 25319637 PMC4336307

[B76] Vulchanova L. Riedl M. S. Shuster S. J. Buell G. Surprenant A. North R. A. (1997). Immunohistochemical study of the P2X2 and P2X3 receptor subunits in rat and monkey sensory neurons and their central terminals. *Neuropharmacology* 36 1229–1242. 10.1016/s0028-3908(97)00126-3 9364478

[B77] Winklbauer R. (1989). Development of the lateral line system in *Xenopus*. *Prog. Neurobiol.* 32 181–206. 10.1016/0301-0082(89)90016-6 2652193

[B78] Xiang Z. Bo X. Burnstock G. (1998). Localization of ATP-gated P2X receptor immunoreactivity in rat sensory and sympathetic ganglia. *Neurosci. Lett.* 256 105–108. 10.1016/s0304-3940(98)00774-5 9853714

[B79] Yegutkin G. G. (2014). Enzymes involved in metabolism of extracellular nucleotides and nucleosides: functional implications and measurement of activities. *Crit. Rev. Biochem. Mol. Biol.* 49 473–497. 10.3109/10409238.2014.953627 25418535

