# Supplementary material for: Comparative Embryonic Spatio-Temporal Expression Profile Map of the Xenopus P2X Receptor Family
Source: Front Cell Neurosci. 2019 Jul 26;13:340. doi: 10.3389/fncel.2019.00340 (PMC6676501; doi:10.3389/fncel.2019.00340)
Supplement: Supplementary file 1 [file Data_Sheet_1.pdf]

Supplemental Data

Protein	*Xenopus laevis*	Protein	*Xenopus tropicalis*
p2rx1.L	XP_018102463.1	p2rx1	XP_002932325.2
p2rx1.S	OCT92343		
p2rx2.L	XP_018115826.1	p2rx2	XP_004910694.1
p2rx2.S	Submitted to Genbank		
p2rx4.L	NP_001082067.1	p2rx4	XP_002937738.2
p2rx5.L	XP_018101534.1	p2rx5	XP_002938288.1
p2rx5.S	XP_018104305.1		
p2rx6.L	OCU02157.1	p2rx6	XP_012824166.
p2rx7.L	NP_001082196.1	p2rx7	XP_017946027.1

Supplemental Table S1A. Accession numbers of the *Xenopus* p2rx sequences

Protein	*Homo sapiens*	*Mus musculus*	*Gallus gallus*	*Danio rerio*	*Takifugu rubripes*
p2rx1	NP_002549.1	NP_032797.3	NP_989850.1	NP_945333.1	XP_011616308.1
p2rx2	NP_057402.1	NP_700449.2	XP_004934477.2	NP_945334.1	XP_003974964.1
p2rx3	NP_002550.2	NP_663501.2	NP_001316145.1	NP_571698.1<br>(p2xr3.1)<br>NP_945337.3<br>(p2xr3.2)	XP_011608945.1<br>XP_003972130.1
p2rx4	NP_001243725.1	NP_035156.2	NP_989622.1	NP_705939.1<br>(p2xr4.1)<br>NP_945338.2<br>(p2xr4.2)	XP_003974770.1
p2rx5	NP_002552.2	NP_201578.2	NP_990079.1	NP_919394.2	XP_003976410.1
p2rx6	NP_005437.2	NP_035158.2	XP_025011318.1	n.a	n.a
p2rx7	NP_002553.3	NP_035157.2	XP_001235163.3	NP_945335.1	XP_003974725.1

Supplemental Table S1B. Accession numbers of the orthologous protein sequences.
n.a. Not applicable as no zebrafish p2rx6 subunit has been described (Kucenas *et al.,* 2003).

Gene	5’-3’ sequence	Localisation	PCR Annealing Temp. Cycle Numbers	Product size (bp)
p2rx1.L	U- CAAAAGGATTCACAAGAAAAGG	E11-12 junction (CDS)	60°C 35 cycles	187 bp
	D- GCAGATCTTCAACGAACTAAAACA	E13 (3'UTR)		
p2rx1.S	U- TGACTGAGTAAGAGCCCAGC	E1 (5'UTR)	63°C 35 cycles	163 bp
	D- TGCCCCATTTTCCCTCTTCT	E1 (CDS+5’UTR)		
p2rx2.L	U- TTGATGAGGTCTGGAAGTTGG	E11-12 junction (CDS)	60°C 34 cycles	178 bp
	D- CGATGTTCTTGCCCCAGT	E12 (CDS)		
p2rx2.S	U- cgctcgaGCATCCTCTCTACACTCTTG	E12 (3’UTR)	59°C 34 cycles	408 bp
	D- cgctcgaGATGAAACAAAGACCACAGG	E12 (3’UTR)		
p2rx4.L	U- CTCTGCCGCGGGCGACTAAT	E12 (3’UTR)	60°C 32 cycles	205 bp
	D- TTTGGCACTGACTCAAATCCTCACAT	E12 (3’UTR)		
p2rx5.L	U- AGTTTGAGGAAGTGAGATCAGC	E11-12 junction (CDS)	57°C 30 cycles	193 bp
	D- TGTGAGTGTGGAAATCACGTA	E12 (3'UTR)		
p2rx5.S	U- CTCCTAACCAGAGACAGGGTAC	E3 (CDS)	61°C 35 cycles	524 bp
	D- TCTCTCCTTGTTATCCAGACGG	E8 (CDS)		
p2rx6.L	U- TGTTATGGGTGGAGTTGTTGC	E7-8 junction (CDS)	63°C 35 cycles	154 bp
	D- TTCCTTCCTTTCTTGGTCCC	E9 (CDS)		
p2rx7.L	U- CCCATATATCCAGAGAGAAAGATTTG	E12-13 junction (CDS)	60°C 35 cycles	174 bp
	D- CAGCACAACTGCTCCTCAAG	E13 (CDS)		
*odc**	U- GGAGCTGCAAGTTGGAGA	E11 (CDS)	60°C 25 cycles	131 bp
	D- TCAGTTGCCAGTGTGGTC	E11-12 junction (CDS)		

Supplemental Table S2. Primer sequences and PCR conditions for the RT-PCR in order to amplify the required genes.
In grey are indicated XhoI restriction site for cloning into pBS-KS.
D: Downstream primer; U: Upstream primer
bp: base pair ; CDS: Coding sequence ; UTR: Untranslated region
* Reference for odc primers : T. Bassez, J. Paris, F. Omilli, C. Dorel, H.B. Osborne (1990) Post-transcriptional regulation of ornithine decarboxylase in *Xenopus laevis* oocytes. *Development* 110 , 955-62.

Marker Gene	RNA polymerase	Linearization	Plasmid Details
p2rx1.L (antisense)<br>*p2rx1.L* (sense)	T7<br>T3	*BamH*I<br>*Kpn*I	p2rx1.L-pBS-SK<br>(CDS+3'UTR ; 2000bp)<br>EST clone (XL078p19)
p2rx1.S (antisense)<br>*p2rx1.S* (sense)	T3<br>T7	*Sal*I<br>*Kpn*I	p2rx1.S-pBS-KS<br>(3'UTR ; 565 bp)<br>Cloned by PCR (st42 cDNA)
p2rx2.L (antisense)<br>*p2rx2.L* (sense)	T3<br>T7	*BamH*I<br>*Kpn*I	p2rx2.L-pBS-KS<br>(3'UTR ; 537 bp)<br>Cloned by PCR (st39 cDNA)
p2rx2.S (antisense)<br>*p2rx2.S* (sense)	T3<br>T7	*Sal*I<br>*Kpn*I	p2rx2.S-pBS-KS<br>(3'UTR ; 408 bp)<br>Cloned by PCR (st24 cDNA)
p2rx4.L (antisense)<br>*p2rx4.L* (sense)	T7<br>Sp6	*EcoR*I<br>*Xba*I	p2rx4.L-pCS111<br>(CDS+3'UTR ; 2000bp)<br>IMAGE clone (8735568)
p2rx5 (antisense)<br>*p2rx5* (sense)	T3<br>T7	*Sal*I<br>*Kpn*I	p2rx5-pBS-KS<br>(CDS (E1-E7) ; 597 bp)<br>Cloned by PCR (st39 cDNA)
p2rx6.L (antisense)<br>*p2rx6.L* (sense)	T3<br>T7	*BamH*I<br>*Kpn*I	p2rx6.L-pBS-KS<br>(CDS (E5-E10) ; 517 bp)<br>Cloned by PCR (st5 cDNA)
p2rx7.L (antisense)<br>*p2rx7.L* (sense)	T7<br>T3	*Kpn*I<br>*Xba*I	p2rx7.L-pBS-KS<br>(CDS; 1400bp)<br>Original clone provided by S.Gründer<br>Insert subcloned into pBS-KS

Supplemental Table 3. In vitro transcription conditions for the in situ probes used in this study.
CDS : Coding Sequence ; E : Exon ; UTR : Unstranslated Region

```
                                                             1
                        ✦                                    ↓
p2rx1.L ------------MGLKMKEKVSNFLFEYDTPRMVLVRDKKVGLTFRMIQVGVLAYIIGWVFVYEKGYQNVDGII-
p2rx1.S ------------MGQKMKEKVSSFLFEYDTPRLVLVQDKKVGLTFRMIQLGVLVYIIGWVFVYEKGYQTVDGII-
p2rx1   ------------MGQKMKEKVSSFLFEYDTPRMVLVRDKKVGLTFRMIQLGVLAYIIGWVFVYEKGYQTVDGII-
p2rx2.L ------MTGCRTRS----MSCIGQLWSYETPKVIVVKNRRLGTIYRLLQLLIIVYFVWYVFVVQKGYQAQETGPE
p2rx2.S ------MTGCRTRS----MSWIGQLWSYETQKVIVVKNRRLGTIYRLLQLLIIVYFVWYVFVVQKGYQAQETGPE
p2rx2   ------MTGCRTRS----MSWIGQLWSYETPKVIVVKNRRLGTIYRLLQLLIVVYFVWYVFVVQKGYQAQETGPE
p2rx4.L -----------MSRDGCCGQAYSCLFDYDTPRIALIKSRKIGLLNRFIQLGILAYVIGWVFIWEKGYQEFDIVV-
p2rx4   MTIPAGERRCAMARDGCCGQVYSCIFDYDTPRIALIKSRKIGFLNRFIQLGILAYVIGWVFIWEKGYQEFDTVV-
p2rx5.L ------------MGQVAWKGLFLSFFDYKTEKYVIAKNKKVGILFRVVQLSILAYLIGWVFVVKKGYQETDTSIQ
p2rx5.S ------------MGQVAWKGLFLSFFDYKTEKYVIAKNKKVGILFRVVQLSLLAYLVGWVFVVKKGYQETDTSIQ
p2rx5   ------------MGQVAWKGLFLSFFDYKTEKYVIAKNKKVGILFRVVQLSILAYLVGWVFVVKKGYQETDTSIQ
p2rx6.L -------------MDAGCAQAQPALLDYKTEKYLLTKNRKVGVIYRFLHLAILCYIIGWVFLAKKGYQDTDSDPH
p2rx6   -------------MDAGCAQAHPALLDYKTEKYVVTKNRKVGVLYRFLHLAILCYTIGWVFLTKKGYQDTDSDPY
p2rx7.L -----------------MTLTLADCFDYSTKKEVRIQSVPLGILKQCITFGVIVFV-CFSLITQKRYQKKDSII-

                                            2                         3
                                            ↓                      *  ↓        *     *
p2rx1.L SSVSVKIKGLAVTDL-PGVGTEIWDAVDYVFPPQ | GDSSFVVMTNFITTADQKMGYCNE | LPDV--APCETNDDCKI
p2rx1.S SSVSVKIKGLAVTDL-PTIGTEIWDAVDYIFPPQ | GDSSFVVMTNFITTADQKMGYCNE | LPDV--APCETNDDCKI
p2rx1   SSVSVKIKSLAVTDL-PSIGTEIWDAVDYVFPPQ | GDSSFVVMTNFITTADQKMGYCNE | LPDV--APCKTNDDCTI
p2rx2.L SAVITKVKGVSTSKG-PRGIKKIWDVAEYVKPPE | GGSVFTIITRIESTKLQTMGTCPE | SRTVPNALCVSDEDCNR
p2rx2.S SAVITKVKGVSTSKG-PRGIKKIWDVAEYVKPPE | GGSVFTIITRIESTKQQTMGTCPE | SRTVPNALCVTDEDCKR
p2rx2   SAVITKVKGVSTSKG-PRGIKKIWDVAEYVKPPE | GGSVFTIITRIESTKLQTMGTCPE | SRTVPNALCVSDEDCNR
p2rx4.L SSVTSKVKGVVVTNT-TELGVKIWDVADYIIPAQ | EENAVFVMTNLILTQNQTQGHCPE | LPET--SFCSKEQPCTP
p2rx4   SSVTSKVKGVAVTNT-SELGVKIWDVADYIIPAQ | EENAFFVMTNLILTQNQTQGYCPE | LPEN--SVCSEKQPCTP
p2rx5.L SSIITKLKGVAFTNT-SELGERLWDVVDYVIPPQ | GENVFFVVTNLIVTPNQRQTMCPE | SFGIPDAACLKNSDCPE
p2rx5.S SSIITKLKGVAFTNT-SELGERLWDVVDYVIPPQ | GENVFFVVTNLIVTPNQRQGTCPE | SFGIPDAECLKNSDCPE
p2rx5   SSIITKLKGVAFTNT-SELGERLWDVVDYVIPPQ | GENVFFVVTNLIVTPNQRQSTCPE | SFGIPDAECSKNSDCPE
p2rx6.L FSIITKLKGVSITKNFRNSQEKIWDVADFVKPSQ | GENVLFLVTNFIATTGQVQRTCAE | NPVNSDARCSENLDCVA
p2rx6   VSIITKLKGVSITKNFRNSQEKIWDVADFVKPSQ | GENVLFLVTNFIATTGQVQRTCPE | NPINLDGRCSENLDCVA
p2rx7.L SSVHTKVKGFADA------HSRIWDTAEYTVPSP | GGDSFFVITNIVKTEGQMQSNCSE | LPSQ-KTICSRDDICKK
p2rx7   SSVHTKVKGFANA------YSRIWDTAEYTVPSP | GGDSFFVVTNIVKTENQRQDNCSE | LPSQ-KTICSRDDVCKK

                 4                                      5                             6
                 ↓     *            *      *            ↓                             ↓
p2rx1.L T-AFRQSQ | GVMTGNCTTYDN--KTKSCQIYAWCPVENDYTVPK | PPLLSQAENFTLFIKNSISFPRHEVTR | QNLVE
p2rx1.S T-AFRQSQ | GVMTGNCTPYDN--KTKTCQIYAWCPVENDYRVPK | PPLLSQAENFTLFIKNSVSFPRHGVTR | QNLVE
p2rx1   T-AFRQSQ | GVMTGNCTPYDN--KTKTCQIYAWCPVENDFNVPK | PPLLSQAENFTLFIKNSISFPRHGVTR | QNLVE
p2rx2.L GEMHILGN | GEQTGRCIIAGR-GSLRTCEIFAWCPLEDETA-IS | ESLQDYVQNFTILIKNNIHFPQFGFSK | GNIED
p2rx2.S GEMHILGN | GEQTGHCIIAGR-GSLRTCEIFAWCPLEDETA-IS | ESLQDYVQNFTILIKNNIHFPQFGFSK | GNIEG
p2rx2   GEMHILGN | GEQTGRCIIAGR-GSLRTCEIYAWCPLEDETA-IS | ESLQDYVQNFTILIKNNIHFPQFGFSK | GNIEG
p2rx4.L GYVGKQSN | GVQTGKCVPYNS--TVKTCEIFAWCPVENDTHVPD | PAFLNGAENFTVLIKNNIWYPKFQVSK | RNILS
p2rx4   GYVGKQSN | GVQTGKCVPYNS--TVNTCEIFAWCPVENDTHVPD | PAFLNGAENFTVLIKNNIWYPKFQAFK | RNILS
p2rx5.L GEPVVAGH | GVKTGRCLKTGAVNATGTCEIFAWCPIEKRKK-PK | IPLLGKAENFTVYIKNSIRFPKFDFSK | TNILE
p2rx5.S GEPVVAGN | GVKTGRCLKTGAVNA--TCEIFAWCPIEKKKN-PK | IPLLGKAENFTVYIKNSIRFPKFDFSK | TNILE
p2rx5   GEPVVAGN | GVKTGRCLKTGAVNATGTCEIFAWCPIEKKKK-PK | TPLLGKAENFTVYIKNSIRFPKFDFSK | TNILE
p2rx6.L GEPVVNGN | GIKTGKCVMFNH--TQSTCEIYGWCPVENESL-LR | KPRLNEAENFTLFIKNVVHFSKFNFSR | ANTLD
p2rx6   GEPVVNGN | GIKTGKCVLFNN--THSTCEIFGWCPVENESL-LR | KPRLNEAENFTLFIKNVIHFSKFNFSR | ANTLD
p2rx7.L GLADPQSN | GIQTGRCINFNN--TLKTCEVSAWCPVESQTT-PV | PAVLESAENFTVLIKNNIHFAAFNFTK | KNILP
p2rx7   GLADPQSN | GIQTGRCINFNS--TLKTCEVSAWCPVESQTT-PV | PAVLESAENFTVLIKNNIHFAAFNFTK | KNILP

                                                            7
                  *          *                              ↓               *         *
p2rx1.L SVTSSYLKKCIYHKETDPLCPVFRLGYIVEQSGRNFSELAYK | GGTISIVIDWQCDLDWPVRYCKPIYEFHGLQD-
p2rx1.S SVTSSYLKKCIYHKKNDPLCPVFRLGYIVKQSGRNFSELAYK | GGTISIVIDWQCDLDWPLRYCKPTYEFHGLQD-
p2rx1   SVTSNYLKKCVYHKENDPLCPVFRLGYIVEQSGRNFSELAYK | GGTISIVIDWQCDLDWPVRYCKPTYEFHGLQD-
p2rx2.L ---QGFLRNCTYHPVLSPSCPIFTVGFIVSEAGANFTELAKK | GGIIGVIINWNCNLDLHPSKCRPEYSFRRLDL-
p2rx2.S ---QGFLRNCNYHPVLSPSCPIFTVGFIVSEAGANFTELAKK | GGIIGVIINWNCNLDLHLSKCRPEYSFRRLDL-
p2rx2   ---QGFLRNCTYHPVLSPSCPIFTVGFIVSEAGANFTELAKK | GGIIGVIINWNCNLDLHPSKCRPEYSFRRLDL-
p2rx4.L NISSSYLKTCQYDKVNHPFCPIFRLGNIVKEAGESFSDMAVQ | GGVMGIQINWNCDLDRKLTYCVPKYSFRRLDNR
p2rx4   NISSSYLKSCQYDKVNHPFCPIFRLGSIVKEAGETFRDMAVQ | GGVMGIQINWDCDLDWKLTYCVPKYSFRRLDNR
p2rx5.L TETESYLKSCRYSN-DDHYCPIFHLGKIVSWAGSEFQRMAIE | GGVIGIQVEWDCNLDKPASECHPNYSFTRLDNK
p2rx5.S TD-ESYLKSCRYSK-DDHYCPIFHLGKIVSWAGSEFQSMAIE | GGVIGIQIEWNCNLDKPASECHPHYSFARLDNK
```

```
p2rx5    TDNGSYLKTCRYSK-DDHYCPIFHLGKIVSWAGSEFQSMAIE|GGVIGIQIEWNCNLDKLASECHPHYSFTRLDNK
p2rx6.L  TSDETYFKNCRYNPVSSPYCPVFQIQEIITQAGQSFEELSVM|GGVVAARIEWKCDLDRPAAECLPQYSFRLQDT-
p2rx6    TSDETYFKNCRYNPFSSPYCPVFQIQEIVTQAGWSFEDLSIM|GGVVAARIEWKCDLDHPAAECLPQYSFRLQDT-
p2rx7.L  N----YNVSCIYDRVKAPLCPIFRLGDILREAGENFSQVAVL|GGVIGIEINWDCDLDPLRYKCEPHYGFRRLDDT
p2rx7    K----YNVSCIYDRVKAPLCPIFRLGDILREAGENFSQVAVL|GGVIGIEINWDCDLDSLRYKCEPHYSFRRLNNK

                       8                              9                       10
                    ▼▽▼↓                ▼             ↓                        ↓
p2rx1.L  --ENKVSQGFNFR|HARYYKE-DGVSKRTLFKVFGIRFDILVNGQ|GGKFDIIPTMTTIGSGIGIFGV|ATVVCDLML
p2rx1.S  --ENKVSQGFNFR|HARYYKE-DGVSKRTLFKVFGIRFDILVNGQ|GGKFNIIPTMTTIGSGIGIFGV|ATIVCDLML
p2rx1    --ENKVSQGFNFR|HARYYKE-DGVSKRTLFKVFGIRFDILVNGQ|GGKFDIIPTMTTIGSGIGIFGV|ATVVCDLML
p2rx2.L  --QNKISSGYNYR|FAKYYNN-NGTETRTLIKVYGIRIDVIVHGQ|AGKFSLIPTIINLATALTSIGV|GSVLCDWIL
p2rx2.S  --QNKISSGYNYR|FAKYYNN-NGTETRTLIKVYGIRIGVIVHGQ|AGKFSLIPTIINLATALTSIGI|GSVLCDWIL
p2rx2    --QNKISSGYNYR|FAKYYNN-NGTETRTLIKVYGIRIDVIVHGQ|AGKFSLIPTIINLATALTSIGV|GSVLCDWIL
p2rx4.L  EIDHNVSPGYNFR|FAKYYKDSNGVESRTLMKVYGIRFDILVFGT|AGKFDIIPTMINIGSGAALFGV|ATVLCDMIV
p2rx4    EIDHNVSPGYNFR|FAKYFKDSNGVESRSLMKVYGIRFDILVFGT|AGKFDIIPTMINIGSGAALFGV|ATVLCDMIV
p2rx5.L  FTEKSISSGYNFR|FAKYYRDAQGNDYRTLIKAYGIRFDIMVNGK|AGKFNIIPTIINIGSGLALMGV|GAFFCDLVL
p2rx5.S  --ERSVSSGYNFR|FAKYYRDAKGNDYRTMFKAYGIRFDIMVTGK|AGKFNIIPTIINIGSGLALMGA|GAFFCDLVL
p2rx5    FTEKSVSSGYNFR|FAKYYRDANGNDYRTLIKAYGIRFDIMVNGK|AGKFNIIPTIINIGSGLALLGA|GAFFCDLVL
p2rx6.L  --------KNNFR|TATYYWDQERKEYRDLFKLYGFRFDISVTGE|ARKFGVVPTAVSLGTGCAFLGA|ATFLCDLIL
p2rx6    --------KNNFR|TATYYWDQERKEYRDLLKLYGFRFDISVTGE|ARKFGLVPTAVSLGTGCAFLGA|ATFLCDLIL
p2rx7.L  VVDESLYPGLNFR|FARYYKNAHGTETRTLIKAYGIRFDIQVYGT|GGQFNLLELALFIGSCLSYFGC|ASFAIDFII
p2rx7    VVDERLYPGLNFR|FARHYKTSDGKDTRTLIKAYGIRFDIQVYGT|GGKFSLFELAIFIGSCLSYFGC|ASLAIDFII

                                         11
                                         ↓
p2rx1.L  LHVLP-------KRNYYKEKKFKQ AK TDQK|D SQEKVEVYTY------------SQNGQQ-LNSSSSENVYDT-QL
p2rx1.S  LHVLP-------KRNYYKEKKFKQ AK REQK|D SQEKEDVSTY------------SQNGQQ-IDSSSSEYVYDT-HL
p2rx1    LHVLP-------KRNYYKEKKFKQ AK TAQK|D SQEKEEV--Y------------SQNGQQ-LNSSSSENVYDT-QL
p2rx2.L  LTFMN-------KDHTYSLRKFDE|VW KLED G TTTAVT---TVSSLQESSC------IPGSPCTFD-EPVYKDKGP
p2rx2.S  LTFMN-------KNHTYSLRKFDE|VW KLED G T--AIT---TVSSLQESSC------ISGSACTLV-EPVYKDMGP
p2rx2    LTFMN-------KDRTYSLRKFDE|VW KLED G NTTAVT---TVSSLQESSC------IPGSPCTLD-EPVYK--GP
p2rx4.L  FHFFK-------KRHYYREKKYKY VE DYDE L|VGSE-C------------------GSN—P           391
p2rx4    FHFFK-------KRHYYREKKYKY VE DYDE L|GGSE-C------------------GSN—P           402
p2rx5.L  LYLIK-------KSNFYRDKKFEE VR|SASK M SLNIKV------------------NGQ--PKSKSHNDLDQV-RL
p2rx5.S  LYLIK-------KSNFYRDKKFED VK|SASK M SLNVKV------------------NGQ--PKSKSQNDLDQV-RL
p2rx5    LYLIK-------KSNFYRDKKFEE VK|SASK M SLNLKV------------------NGK--PKSKSHNDLDQI-KL
p2rx6.L  LYLDK-------KASFYRSCKYEE|VK PPKN Q QTIQET------------------RNE              383
p2rx6    LYLDK-------KASFYRSCKYEE|AK PPKR Q QNIQET------------------NNK—E            384
p2rx7.L  GRYNSCCCNAKSVLKYYDDRKYET IPGPSVS L|AHLKAHLKFVSFVDKEDILMVDQKLKGSLQLASGPYIQ|RE-RF
p2rx7    GLYKPCCCNAKSVLKYYNDRKYEM VPGPAVP L|CFSKSQLKFVSFADEEDILMVDNNLKDSLQFASGRYIQ|RE-RF

p2rx1.L  NT-YNSC                                         412
p2rx1.S  NT-YK                                           410
p2rx1    NT-YKSC                                         410
p2rx2.L  AMEFTIIQDRLGQ--------EHRDGILPPPGS               438
p2rx2.S  AVEFTIIHDGLGQ-----------DGILPPPGS               433
p2rx2    AVEFTIIQDRLEQ--------EQQDGIFPPPGS               436
p2rx4.L
p2rx4
p2rx5.L  QP-LEA                                          412
p2rx5.S  QP-LEA                                          407
p2rx5    QS-VEA                                          412
p2rx6.L
p2rx6
p2rx7.L  ADTKAKCKDSHKQDENEMRLIKGRSAMLPPAWCKCNKCINTTHLEEQLCCRLEEGECITDTKMFNSLVLNRESLE
p2rx7    AYTEANCKDSHKQNESEMRLIKGSSTTLPPAWCKCNKCIDVTQPEEQLCCRLGEGQCITDTKIFNYLVLNKEALE

p2rx7.L  YAFQYDNPLSKTPISKEHLRYYAKQKYVEWRFGCRKYMLNFAVIPNCCKTAIETCNLQTEGP            553
p2rx7    YAFQYDNPLSKTPES-EDLKCYAKQKYIEWRFRCRRYMLDFAVIPSCCKNAIETCNLQTQHPSGALYLPPTHGMC 565
```

Supplemental Figure S1. Protein sequence alignment of *Xenopus* p2x receptor subunits.

Alignment of the *Xenopus laevis* and *tropicalis* p2rx proteins was performed using CLUSTALW. The Genbank accession numbers are given in the Table S1A. The size of the proteins (AA numbers) is indicated at the end of the protein sequences. Spaces in the sequences are indicated by a dash. The exons boundaries (numbered 1 to 11) are indicated by the green vertical lines and arrow (except for the non-conserved boundary 11) and the residues straddling those boundaries, when existing, are indicated in green. Residues conserved in the 15 sequences are indicated in bold red. Residues conserved in 5 over the 6 sub-families are indicated in blue. Asterisks indicate the 10 conserved cysteines involved in disulfure bonds (North, 2002). Triangles indicate the 8 residues involved in ATP binding (Habermarcher *et al.,* 2016), the black ones the residues conserved in all sequences and the white one the residue not conserved in p2x2 subunits. Double line indicates the consensus Tyr-XXX-Lys (YXXXK) motif for surface targeting (Chaumont *et al.*, 2004). The star indicates the conserved Threonine of the Thr-X-Arg/Lys (TXR/K) PKC phosphorylation site (indicated by the dotted line) involved in desensitization of P2X receptor (Boué-Grabot et *al.*, 2000b). Black lines above sequences indicate the two putative transmembrane domains, predicted with ExPASy TMPred tool.
